# Vitamin K Status Based on K1, MK-4, MK-7, and Undercarboxylated Prothrombin Levels in Adolescent and Adult Patients with Cystic Fibrosis: A Cross-Sectional Study

**DOI:** 10.3390/nu16091337

**Published:** 2024-04-29

**Authors:** Patrycja Krzyżanowska-Jankowska, Jan Nowak, Marta Karaźniewicz-Łada, Małgorzata Jamka, Eva Klapkova, Szymon Kurek, Sławomira Drzymała-Czyż, Aleksandra Lisowska, Irena Wojsyk-Banaszak, Wojciech Skorupa, Jarosław Szydłowski, Richard Prusa, Jarosław Walkowiak

**Affiliations:** 1 Department of Pediatric Gastroenterology and Metabolic Diseases, Poznan University of Medical Sciences, Szpitalna Street 27/33, 60-572 Poznan, Poland; jannowak@ump.edu.pl (J.N.); mjamka@ump.edu.pl (M.J.); skurek@ump.edu.pl (S.K.); jarwalk@ump.edu.pl (J.W.); 2 Department of Physical Pharmacy and Pharmacokinetics, Poznan University of Medical Sciences, Rokietnicka Street 3, 60-806 Poznan, Poland; mkaraz@ump.edu.pl; 3 Department of Medical Chemistry and Clinical Biochemistry, 2nd Faculty of Medicine, Charles University, V Úvalu 84, 150 06 Prague, Czech Republic; eva.klapkova@fnmotol.cz (E.K.); richard.prusa@fnmotol.cz (R.P.); 4 Department of Bromatology, Poznan University of Medical Sciences, Rokietnicka Street 3, 60-806 Poznan, Poland; drzymala@ump.edu.pl; 5 Department of Pediatric Diabetes, Auxology and Obesity, Poznan University of Medical Sciences, Szpitalna Street 27/33, 60-572 Poznan, Poland; alisowska@ump.edu.pl; 6 Department of Pneumonology, Allergology and Clinical Immunology, Poznan University of Medical Sciences, Szpitalna Street 27/33, 60-572 Poznan, Poland; iwojsyk@ump.edu.pl; 7 Department of Lung Diseases, Institute for Tuberculosis and Lung Diseases, Plocka Street 26, 01-138 Warsaw, Poland; w.skorupa@igichp.edu.pl; 8 Department of Pediatric Otolaryngology, Poznan University of Medical Sciences, Szpitalna Street 27/33, 60-572 Poznan, Poland; jszydlow@ump.edu.pl

**Keywords:** vitamin K2, vitamin K1, cystic fibrosis, pancreatic insufficiency, gastroenterology, liquid chromatography

## Abstract

The available evidence on vitamin K status in cystic fibrosis (CF) is scarce, lacking data on vitamin K2 (menaquinones—MK). Therefore, we assessed vitamin K1, MK-4 and MK-7 concentrations (LC-MS/MS) in 63 pancreatic insufficient and modulator naïve CF patients, and compared to 61 healthy subjects (HS). Vitamin K1 levels did not differ between studied groups. MK-4 concentrations were higher (median <1st–3rd quartile>: 0.778 <0.589–1.086> vs. 0.349 <0.256–0.469>, *p* < 0.0001) and MK-7 levels lower (0.150 <0.094–0.259> vs. 0.231 <0.191–0.315>, *p* = 0.0007) in CF patients than in HS. MK-7 concentrations were higher in CF patients receiving K1 and MK-7 supplementation than in those receiving vitamin K1 alone or no supplementation. Moreover, vitamin K1 concentrations depended on the supplementation regime. Based on multivariate logistic regression analysis, we have found that MK-7 supplementation dose has been the only predictive factor for MK-7 levels. In conclusion, vitamin K1 levels in CF are low if not currently supplemented. MK-4 concentrations in CF patients supplemented with large doses of vitamin K1 are higher than in HS. MK-7 levels in CF subjects not receiving MK-7 supplementation, with no regard to vitamin K1 supplementation, are low. There do not seem to be any good clinical predictive factors for vitamin K status.

## 1. Introduction

Vitamin K levels mainly depend on intake and absorption. Vitamin K is absorbed in the terminal ileum and arrives in the lymphatic system, with bile salts and pancreatic lipase being essential in this process [1,2]. Exocrine pancreatic insufficiency and the absence of lipase occur very early in cystic fibrosis (CF) and are present in about 85% of patients by the end of the first year of life. There is also abnormal viscosity, decreased flow, and increased concentration of bile components in CF, thereby affecting the function of bile salts [3]. Bile salt secretion may also be affected in asymptomatic fibrosing liver disease in as many as 25–30% of CF patients [4]; therefore, CF patients are at risk of developing fat-soluble vitamin deficiency, including vitamin K [2,5,6]. In CF patients, vitamin K deficiency has appeared despite its supplementation and despite the use of a large dose of vitamin K1, functional markers of protein carboxylation have not normalized in many cases [7].

The assessment of vitamin K body resources is challenging, as there is no standard laboratory test to address this issue [8]. Prothrombin time and international normalized ratio (INR) are not adequately sensitive and can be used only when undercarboxylated coagulation factors exceed 50% [9]. The available data regarding vitamin K status in CF patients are mainly based on the assessment of undercarboxylated prothrombin (PIVKA-II—protein induced by vitamin K absence), undercarboxylated osteocalcin (u-OC), or vitamin K1 (phylloquinone) levels [9,10,11,12,13,14,15,16,17,18]. However, there is no evidence evaluating vitamin K2 (menaquinone—MK) concentrations, which are potentially important since ingested vitamin K1 is metabolised to MK-4 [19].

The present study assessed the vitamin K status based on the estimation of vitamin K1 and PIVKA-II levels, as well as MK-4 and MK-7 concentrations, in adolescent and adult CF patients. We hypothesised that vitamin K status in CF subjects does not differ from healthy controls.

## 2. Materials and Methods

The study group comprised 63 CF modulator naïve patients aged 16.3 to 46.5 years (median age 23.1 years) recruited from the Department of Pediatric Gastroenterology and Metabolic Diseases, Poznan University of Medical Sciences Poznan, Poland and Department of Lung Diseases, Institute for Tuberculosis and Lung Diseases, Warsaw, Poland. The recruitment and data collection process started in March 2018 and ended in September 2018. The inclusion criteria included CF diagnosed according to European Guidelines [20,21] and pancreatic insufficiency (faecal elastase-1 < 100 μg/g of stool) [22,23]. Individuals were excluded if they had grave medical conditions concerning poor nutritional status, end-stage pulmonary disease, and forced expiratory volume in one second % (FEV1) <20%.

Sixty-one healthy adults aged 18.5 to 29.4 years (median age 22.5 years) not receiving vitamin K constituted the comparative group. To eliminate potential sources of bias, the control group had similar age, gender, weight, and body height distribution to the CF group. Nutritional status (body weight, height, and body mass index—BMI) was assessed in both groups (Table 1).

Genotypes were considered and clinical expression of CF disease (lung function: spirometry, permanent, or periodic *Pseudomonas aeruginosa* colonisation) was estimated in all CF patients. Additionally, information about coexisting diseases (diabetes, cystic fibrosis-associated liver disease—CFLD) was collected. Permanent or periodic *Pseudomonas aeruginosa* colonisation was detected in 40 (63.5%) CF patients, CFLD and diabetes in 29 (46.0%) and 11 (17.5%) CF subjects, respectively. The *CFTR* genotypes in CF participants were as follows: F508del/F508del (n = 33), F508del/- (n = 5), F508del/CFTRdele2,3(21kb) (n = 3), F508del/1717-1G>A (n = 3), F508del/2143delT (n = 2), F508del/2183AA>G (n = 2), F508del/N1303K (n = 2), F508del/G27V (n = 1), F508del/G551D (n = 1), F508del/R553X (n = 1), F508del/R851X (n = 1), F58del/C525X (n = 1), F508del/1078delT (n = 1), F508del/3849+10kbC>T (n = 1), 2185insA/D1152H (n = 1), S1196X/Q138X (n = 1), -/- (n = 4).

Thirty-seven CF subjects were receiving vitamin K1 (Vitacon, Polpharma; median dose [1st–3rd quartile]: 2.9 mg/day [2.1–5.0]). Nineteen subjects were supplemented with vitamin K1 (median dose [1st–3rd quartile]: 0.5 mg/day [0.5–1.0]) and MK-7, of which, 17 patients received a complex preparation (Cystisorb, Norsa Pharma) containing 500 µg of vitamin K1 and 100 µg of MK-7 (10 patients—1 tablet and 7 patients—2 tablets) and 2 patients were supplemented with Vitacon and Cystisorb (1 tablet and 2 tablets, respectively).

Blood samples were collected from all participants after an overnight fast (without eating or drinking for 8 h) in the early morning. Vitamin K1, MK-4, and MK-7 concentrations were estimated in the serum using a Nexera UPLC liquid chromatograph coupled with a triple quadrupole mass spectrometer (LCMS-8030, Shimadzu, Kyoto, Japan). The serum samples for analysis were prepared based on the method of Dunovska et al. (2019) [24]. PIVKA-II concentrations were assessed using an enzyme-linked immunosorbent assay kit (Cusabio, Wuhan, China). The concentrations of individual vitamin K isoforms and PIVKA-II were assessed in both groups using the same analytical methods.

Assuming a significance level (α) of 0.05 and a power (β) of 0.2, with a 20% difference in predicted means and an expected standard deviation (SD) equal to 35% of the mean, it was recommended to recruit 36 subjects in each group to achieve a power of 80%. Accounting for a 20% dropout rate, the total sample size should be 88 subjects. These calculations were conducted using G*Power software (version 3.1.9.2, University of Kiel, Kiel, Germany). The predicted mean and expected standard deviation were determined for the vitamin K1 levels obtained from our pilot study.

The Shapiro–Wilk test was used to check the normality of the data distribution. Due to the lack of normal distribution, the data are presented as medians with interquartile ranges. The Mann–Whitney U test was used to compare the clinical parameters in CF patients and healthy adults, as well as CF individuals with the lowest and the highest concentrations of vitamins K1, MK-4, and MK-7. The effect size using Cohen’s D index was calculated to compare vitamin K1, MK-4, and MK-7 concentrations in CF patients and HS. The Kruskal–Wallis test by ranks (followed by post hoc Dunn’s test) was used to compare CF patients with vitamin K1 supplementation receiving K1 and MK-7 and CF subjects without supplementation. Spearman correlation between K1, MK-4, MK-7 concentration, and clinical parameters in study subgroups (CF patients receiving only vitamin K1; receiving vitamin K1 together with MK-7; and not receiving any supplementation) were also assessed. Moreover, Fisher’s exact test was used to determine differences between the subgroups with the lowest and the highest vitamin K1, MK-4, and MK-7 levels concerning qualitative parameters (gender, CFLD, diabetes, *Pseudomonas aeruginosa* colonisation, *CFTR* gene mutation, and vitamin MK-7 supplementation). Univariate logistic regression analysis was subsequently performed to assess the relationship between clinical parameters and serum K1, MK-4, and MK-7 concentrations in CF patients. To confirm the relationship between vitamin K doses (K1, MK-7) and its isoform levels, parameters that were used in the univariate analysis (for which *p* < 0.1) were entered into a multivariate logistic regression analysis. The level of significance was set at *p* < 0.05. Statistical analyses were performed using STATISTICA 13, Copyright 1984–2017 TIBCO Software Inc. The study was written according to the STROBE guidelines [25].

## 3. Results

**Figure 1:**
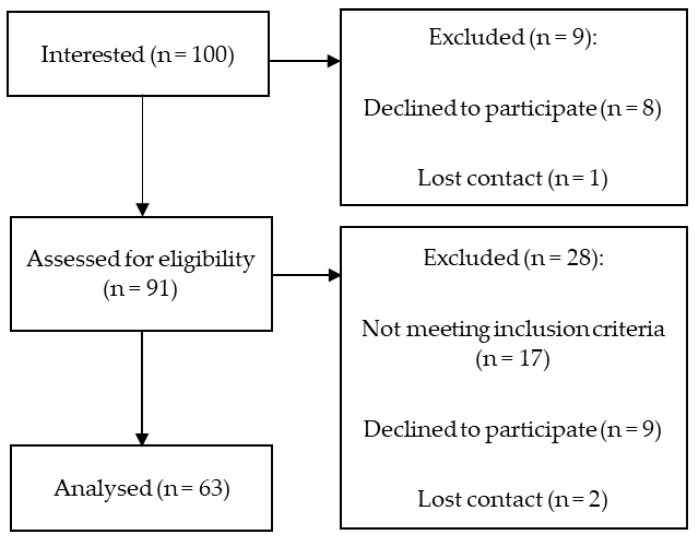
Study flowchart for CF group.

**Figure 2:**
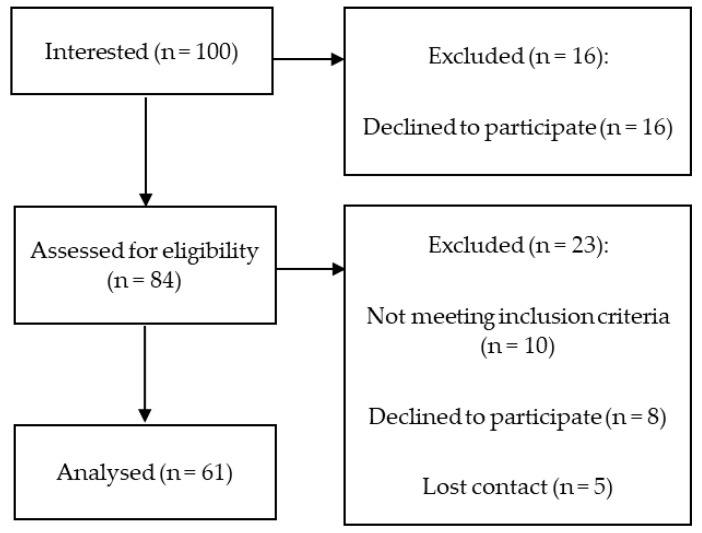
Study flowchart for control group.

Overall, 100 pancreatic insufficient CF patients expressed an interest in participating in the study, of which, 63 subjects were enrolled and completed the study. Seventeen participants were excluded because they did not meet the inclusion criteria, 17 patients declined to participate, and we lost contact with 3 CF patients (Figure 1). A total of 100 healthy controls were interested in participating in the study, of which 10 healthy adults were excluded because of a BMI > 24.9 [kg/m^2^], 24 withdrew, and we lost contact with 5 participants, so the control group comprised 61 subjects (Figure 2).

The CF patients had significantly higher MK-4 (Cohen’s D index-1.42) and lower MK-7 concentrations (Cohen’s D index-0.11) than the healthy adults. However, vitamin levels were significantly different in particular subgroups of CF patients. MK-4 concentrations did not differ in CF patients receiving only vitamin K1, both K1 and MK-7, or those without supplementation. However, MK-7 concentrations were significantly higher in CF patients receiving K1 and MK-7 supplementation simultaneously than in CF subjects receiving vitamin K1 alone or no supplementation. Moreover, vitamin K1 concentrations depended on the supplementation regime. The nominally lowest concentration [median (1–3 quartile): 0.093 (0.066–0.298) ng/mL] was found in the subgroup not taking vitamin K supplementation, but it is not possible to demonstrate statistical significance in post hoc tests due to the small sample number. Furthermore, there were no significant differences in PIVKA-II concentrations between all CF adults and healthy controls, or between CF patients receiving or not receiving vitamin K supplementation (Table 2). The effect size for vitamin K1, MK-4, and MK-7 concentrations in particular subgroups of CF patients has been presented in Table 3.

Serum vitamin K1 levels negatively correlated with body weight in all CF individuals. In CF patients receiving only vitamin K1, a positive correlation was found between vitamin K1 concentration and vitamin K1 dosage, but there were no statistically significant correlations in patients who were supplemented both with K1 and MK-7 (Table 4).

The comparison of CF patients with the lowest (1st quartile) and the highest (3rd quartile) vitamin K1, MK-4, and MK-7 concentrations revealed few differences. CF patients with the highest vitamin K1 concentration were supplemented with a higher vitamin K1 dose. Moreover, higher vitamin MK-7 levels were observed more frequently in CF patients receiving supplementation (Table 5 and Table 6).

Based on univariate and multivariate logistic regression analysis, the MK-7 supplementation dose was found to be a predictive factor for MK-7 levels in CF patients. This determinant explained about 20% of the variability in MK-7 levels (Table 7 and Table 8). However, for PIVKA-II, K1, and MK-4 as dependent variables, the statistical significance of individual models was not achieved.

## 4. Discussion

The vitamin K status in adolescent and adult CF patients was documented for the first time based not only on the estimation of vitamin K1 and PIVKA-II levels, but also on MK-4 and MK-7 concentrations. K1 and PIVKA-II levels did not differ between CF and healthy subjects, whereas MK-4 concentrations were significantly higher and MK-7 levels lower in CF patients than in healthy controls. However, vitamin K concentrations in CF patients were associated with K1 and MK-7 supplementation. Vitamin K1 levels were low if patients did not take supplements (more precisely, they were not taking the vitamin when assessing its body resources). MK-4 concentrations in CF patients supplemented continuously or in the recent past were higher than in healthy controls, and MK-7 levels were dramatically lower in CF subjects not receiving MK-7 supplementation. Interestingly, the PIVKA-II levels were not as high as previously documented [7,26]. Vitamin K1 deficiency seems to be a common problem in CF [7,9,12,15]. Insufficient levels of vitamin K can cause impaired blood coagulation and bone formation. However, CF patients can occasionally present bleeding disorders. Therefore, supplementation and monitoring of vitamin K1 status are very important [2]. For many years INR or prothrombin time have been used for the assessment of vitamin K status. Subsequently, PIVKA-II was considered as a marker of vitamin K deficiency. There are some available data on vitamin K1 levels in CF patients [9,10,11,12,13,14,15,16,17,18]. However, there has been no evidence evaluating vitamin K2 concentrations, neither MK-4 nor MK-7 in CF, to date.

Vitamin MK-7, such as MK-4 and vitamin K1, is a cofactor of enzyme γ-carboxylase, which plays an important role in converting inactive vitamin K-dependent proteins (osteocalcin and matrix Gla protein) to their active form [27,28]. Vitamin MK-7 has health-beneficial effects in osteoporosis [29,30,31], cardiovascular disease (inhibition of vascular calcification) [32,33,34], inflammation [35], cancer (anticancer effects on various cell lines such as hepatocellular carcinoma, leukemia, cholangiocarcinoma, ovarian cancer, pancreatic cancer, and colorectal cancer) [36,37,38,39,40,41], Alzheimer’s disease [42], diabetes [43], and peripheral neuropathy [44,45]. Moreover, this isoform of vitamin K2 is a modulator of different genes in the diseases listed above [28]. In our study, the lower MK-7 levels in CF patients compared to controls may be due to a higher risk of deficiency of fat-soluble vitamins in CF, including vitamin K. The disturbed mechanism of intestinal absorption due to pancreatic insufficiency and bile salt deficiency, liver disease, bowel resection, antibiotic treatment, and inadequate dietary intake can lead to vitamin K deficiency in CF [5,6,46,47]. In addition, it is mandatory to take vitamin K1, not K2, in many other countries [6,48,49,50]. However, the Cohen D index of 0.11 indicates that despite the statistically significant (*p* = 0.0007) difference in MK-7 concentrations between healthy and CF patients, the effect size is limited. On the other hand, the Cohen index values of 0.975 and 0.254 suggest that differences in levels of this vitamin between CF patients receiving MK7 supplementation (and K1) and, respectively, temporarily not receiving vitamin K supplements or receiving vitamin K1 only, are more meaningful. It is worth defining in the future whether MK7 doses, similarly to K1 doses, should be larger in CF. PIVKA-II concentrations did not differ between CF patients and healthy controls, indicating a better vitamin K status attributed to generally better clinical status and high vitamin K doses. Data regarding PIVKA-II levels in patients with CF compared to healthy controls are limited. Differences in the size of the groups, how CF patients were divided into subgroups, the participant’s age, and the supplement vitamin K doses make it difficult to compare the available results [51,52]. Rashid et al. investigated PIVKA-II levels in 98 CF patients aged 0.6−45.3 years and 62 healthy individuals aged 1−45 years, and subdivided the CF patients into three groups: CF with pancreas insufficiency, CF with pancreas sufficiency, and CF with pancreas insufficiency and liver disease. All control subjects had normal PIVKA-II levels below 3 µg/L, with the highest PIVKA-II levels found in CF patients with pancreatic insufficiency [51]. In contrast, van Hoorn et al. compared 20 CF patients on three different doses of vitamin K supplementation (0 mg/day, <0.25 mg/day, and ≥1 mg/day) with 19 healthy controls and did not observe any significant differences in PIVKA-II levels between healthy people and CF patients taking the highest dose of vitamin K [52]. Although we did not document differences in PIVKA-II levels between selected subgroups, it is worth mentioning that undercarboxylated prothrombin concentrations were higher in CF patients not receiving (n = 7) vitamin K than those receiving (n = 56) supplementation (3.37 <2.07–3.97> vs. 1.48 <0.74–2.90> ng/mL, *p* = 0.0821).

Interestingly, significantly higher levels of MK-4 were observed in CF patients, most of whom took vitamin K1, compared to healthy controls. The effect size for this phenomenon is very large, as evidenced by the Cohen’s D index of 1.42. This may be due to the previously described conversion of orally administered vitamin K1 to MK-4 [53,54]. It is unclear whether vitamin K1 is absorbed and delivered to tissues in its original form and then converted to MK-4, or whether it undergoes side chain cleavage in the intestine to menadione and is converted to MK-4 after delivery to tissues [55]. The mechanism of this interconversion was first described by Hirota et al., who reported that the deuterium-labelled form of vitamin K1 (PK-d7) administered orally to rats was converted to the quinone form of menadione (MD-d7) in the intestine under the influence of the phytyl side-chain cleaving enzyme. This form of menadione was transported to the tissues via the mesenteric lymphatic system and blood circulation, and was converted to the hydroquinone form (menadiol) by a redox enzyme. Finally, menadiol was converted to MK-4-d7 by the enzyme UbiA prenyltransferase domain-containing protein 1 (UBIAD1) [19]. Unfortunately, there is no data describing how vitamin MK-4 is released from the tissues into the bloodstream. Considering the obtained results, it seems that large supplementary doses of vitamin K1 in CF patients resulted in increased MK-4 levels. Paradoxically, MK-4 levels in CF patients (temporarily) without any supplementation are not lower, which may be related to long tissue retention, but this requires further investigation. It seems that the functional effect of vitamin K supplementation may demand higher blood levels in CF patients than in HS.

Vitamin K1 concentrations were negatively correlated to body weight (rho = −0.2556, *p* = 0.0431) in all CF individuals. Positive correlations were observed between vitamin K1 levels and its dose in CF subjects taking only vitamin K1 supplementation (rho = 0.3788, *p* = 0.0208). However, there were no correlations in subgroups receiving vitamin K1 alone or additional MK-7. There is no available data to evaluate the correlations between vitamin K1 and K2 in CF with most studies describing the analysis of correlations between various clinical parameters (including biochemical parameters of bone turnover) and markers of vitamin K body resources such as PIVKA-II and/or u-OC percentage [12,13,14,56,57].

CF patients with the highest vitamin K1 levels received higher doses of vitamin K1 than those with the lowest concentrations and typically, nonsupplemented CF patients had lower vitamin MK-7 concentrations similar to our previous study [7], in which we observed a higher incidence of vitamin K deficiency in patients not taking vitamin K1 supplements. However, vitamin K deficiency was described by PIVKA-II levels and u-OC percentage [7].

The simultaneous impact of independent variables (vitamin K1 and MK-7 doses) on the dependent variable, such as MK-7 concentration, was assessed using multivariate logistic regression analysis, showing that the lower MK-7 dose is a predictor factor of low vitamin MK-7 concentration in CF subjects. This determinant explained about 20% of the variability in MK-7 levels. However, for PIVKA-II, K1, and MK-4 as dependent variables, the statistical significance of individual models was not achieved. In our previous studies, we searched for good predictors of vitamin K body resources based on the PIVKA-II level and u-OC percentage in CF individuals [7,26,58]. In a large cohort of supplemented and nonsupplemented CF patients, we found that liver involvement, diabetes, and glucocorticoid therapy were potential risk factors for vitamin K deficiency based on PIVKA-II levels, and the vitamin K dose was a predictive factor for u-OC percentage [7]. In CF patients who were not supplemented with vitamin K, a gamma-glutamyl transferase was defined as a potential determinant of PIVKA-II concentration [58]. In a prospective cohort study involving CF patients with liver cirrhosis, the dose of vitamin K and F508del mutation were potentially defined as determinants of u-OC percentage [20]. Dougherty et al. (2010) reported that vitamin K supplementation, 25(OH)D3, the age in men, and vitamin K supplementation only in women were significant predictors of % u-OC [57]. Nicolaidou et al. (2006) also showed that vitamin K supplementation was a predictor of u-OC, c-OC, carboxy-terminal propeptide of type I procollagen, and amino-terminal propeptide of type I procollagen concentrations in CF subjects [14]. Overall, the data concerning the predictor factors of vitamin K status, especially measured as particular isoforms, is limited. This study has certain limitations that warrant consideration. Firstly, the assessment of vitamin K intake from the diet was not included in our evaluation. Secondly, the study sample size was relatively small. However, it is important to note that this study represents the first pilot cross-sectional investigation that not only compared vitamin K1 and PIVKA-II levels, but also examined and compared MK-4 and MK-7 concentrations between CF participants and healthy subjects. Consequently, further research is essential to validate our findings. It is worth defining whether vitamin K supplementation should comprise a combination of vitamin K1 and MK7 (or maybe vitamin K2 alone), as well as defining potential doses. An interesting direction for future research will be investigating the long-term effects of vitamin MK-7 supplementation on clinical outcomes in CF patients, including cardiovascular risk. Furthermore, it is crucial to acknowledge that our CF population comprised individuals who were modulator naïve, thus, caution should be exercised in generalising the results to CF patients undergoing modulator therapy. Triple therapy (elexacaftor/tezacaftor/ivacaftor) seems to improve fat-soluble vitamin status [59]. On the other hand, it influences nutritional status towards overnutrition [60]. Therefore, future studies should focus on assessing vitamin K levels in CF patients treated with CFTR modulators.

## 5. Conclusions

In conclusion, vitamin K1 levels in CF are low if not currently supplemented, and MK-4 concentrations in CF patients supplemented with large doses of vitamin K1 are higher than in healthy subjects. Interestingly, MK-7 levels in CF subjects not receiving MK-7 supplementation, with no regard to vitamin K1 supplementation, are low. Vitamin K status in CF seems to be closer to normal than in the past, but there is still a lack of good clinical predictive factors for its assessment. Only vitamin MK-7 dosing has been documented as a potential risk factor for its deficiency. Further studies are warranted to confirm the results, especially those related to vitamin K2.

## Figures and Tables

**Table 1:** Clinical characteristics of patients with CF and healthy controls.

Clinical Parameters Median (1st–3rd Quartile)		CF Group (N = 63)	Control Group (N = 61)	*p*
Gender	Female	40 (63.5%)	41 (67.2%)	0.6634
	Male	23 (36.5%)	20 (32.8%)	
Age [years]		23.1 (19.4−29.4)	22.5 (21.4−23.4)	0.4656
Body weight [kg]		59.0 (51.5−66.5)	60.0 (55.0−68.5)	0.1844
Body height [cm]		168 (161−174)	168 (163−176)	0.5402
BMI [kg/m^2^]		20.9 (19.7−22.2)	21.6 (20.4−22.7)	0.0829

CF—cystic fibrosis, BMI—body mass index.

**Table 2:** Comparison of K1, MK-4, MK-7, and PIVKA-II concentrations between CF and healthy adults.

Median (1st–3rd Quartile)	CF all (N = 63)	CF with K1 Supplementation (N = 37)	CF with K1 and MK-7 Supplementation (N = 19)	CF without Any Supplementation (N = 7)	Healthy Adults (N = 61)	*p*
K1 [ng/mL]	0.315 (0.169−0.532)	0.407 (0.225–0.573)	0.270 (0.145–0.396)	0.093 (0.066–0.298)	0.274 (0.203–0.387)	0.3526 ^a^ 0.0453 ^b^
MK-4 [ng/mL]	0.778 (0.589−1.086)	0.782 (0.600–1.163)	0.748 (0.587–1.029)	0.671 (0.533–0.840)	0.349 (0.256–0.469)	<0.0001 ^a^ 0.4219 ^b^
MK-7 [ng/mL]	0.150 (0.094–0.259)	0.140 ** (0.095–0.188)	0.259 *^,^** (0.178–0.464)	0.093 * (0.068–0.137)	0.231 (0.191–0.315)	0.0007 ^a^ 0.0063 ^b^
PIVKA-II [ng/mL]	1.78 (0.86−3.25)	1.16 (0.73–2.18)	2.47 (1.38–3.67)	3.37 (2.07–3.97)	1.63 (0.74–2.64)	0.5671 ^a^ 0.0606 ^b^
Vitamin K1 [mg/kg/day]	0.03 (0.01–0.06)	0.06 (0.03–0.08)	0.01 (0.01–0.02)	-	-	<0.0001 ^c^
MK-7 [µg/kg/day]	0 (0–1.57)	-	2.08 (1.74–3.03)	-	-	-

CF—cystic fibrosis, PIVKA-II—undercarboxylated prothrombin. ^a^ CF all vs. healthy adults, ^b^ CF with K1 supplementation vs. CF with K1 and MK-7 supplementation vs. CF without supplementation. ^c^ CF with K1 supplementation vs. CF with K1 and MK-7 supplementation, *^,^** statistical significance (*p* < 0.05) in Dunn’s multiple comparison test (post hoc Dunn’s test).

**Table 3:** The effect size for vitamin K1, MK-4, and MK-7 concentrations in particular subgroups of CF patients.

Mean ± SD	CF with K1 Supplementation (N = 37)	CF with K1 and MK-7 Supplementation (N = 19)	Cohen’s D index
**K1 [ng/mL]**	0.986 ± 1.769	0.556 ± 1.009	0.299
**MK-4 [ng/mL]**	0.901 ± 0.450	0.899 ± 0.525	0.004
**MK-7 [ng/mL]**	0.294 ± 0.805	0.464 ± 0.497	0.254
	**CF with K1 Supplementation** **(N = 37)**	**CF without any Supplementation** **(N = 7)**	**Cohen’s D index**
**K1 [ng/mL]**	0.986 ± 1.769	0.245 ± 0.293	0.584
**MK-4 [ng/mL]**	0.901 ± 0.450	0.636 ± 0.257	1.711
**MK-7 [ng/mL]**	0.294 ± 0.805	0.115 ± 0.095	0.312
	**CF with K1** **and MK-7 Supplementation** **(N = 19)**	**CF without any Supplementation** **(N = 7)**	**Cohen’s D index**
**K1 [ng/mL]**	0.556 ± 1.009	0.245 ± 0.293	0.419
**MK-4 [ng/mL]**	0.899 ± 0.525	0.636 ± 0.257	0.636
**MK-7 [ng/mL]**	0.464 ± 0.497	0.115 ± 0.095	0.975

**Table 4:** Spearman correlations between K1, MK-4, MK-7 concentrations and clinical parameters in CF patients.

Clinical Parameters	Study Subgroups	K1 [ng/mL]		MK-4 [ng/mL]		MK-7 [ng/mL]	
		Rho	*p*	rho	*p*	rho	*p*
Age [years]	I	0.1241	0.3325	0.0988	0.4411	−0.0678	0.5976
	II	0.2160	0.1991	0.2067	0.2198	0.1123	0.5082
	III	−0.0650	0.7916	−0.1027	0.6756	−0.0720	0.7696
Body weight [kg]	I	−0.2556	0.0431	−0.0664	0.6053	−0.1632	0.2013
	II	−0.1293	0.4458	−0.2276	0.1756	−0.2004	0.2344
	III	−0.3038	0.2061	0.2283	0.3472	−0.1466	0.5492
Body height [cm]	I	−0.1677	0.1890	0.0179	0.8896	−0.0576	0.6537
	II	−0.0084	0.9605	−0.0970	0.5679	−0.0714	0.6742
	III	−0.3008	0.2108	0.2348	0.3332	0.0897	0.7149
BMI [kg/m^2^]	I	−0.2007	0.1147	−0.0896	0.4850	−0.2240	0.0776
	II	−0.1011	0.5515	−0.2382	0.1558	−0.2344	0.1627
	III	−0.1140	0.6420	0.1228	0.6165	−0.2754	0.2537
FEV1 [s]	I	0.0075	0.9538	0.1099	0.3950	−0.0401	0.7571
	II	0.0302	0.8590	0.0977	0.5650	−0.1945	0.2487
	III	0.0279	0.9125	0.0386	0.8752	−0.1203	0.6238
Vitamin K1 dose [mg/kg/day]	II	0.3788	0.0208	0.2619	0.1174	0.1203	0.4781
	III	−0.0053	0.9829	−0.0754	0.7589	−0.0579	0.8139
MK-7 dose [µg/kg/day]	III	0.1580	0.5184	0.0570	0.8166	0.1071	0.6626
PIVKA-II [ng/mL]	I	−0.0877	0.4943	0.0383	0.7658	−0.1018	0.4274
	II	−0.0598	0.7248	0,0149	0.9301	−0.1494	0.3776
	III	0.2982	0.2149	0.1614	0.5092	0.0351	0.8866

BMI—body mass index, FEV1—forced expiratory volume in one second, PIVKA-II—undercarboxylated prothrombin. I—all CF patients, II—CF patients receiving only vitamin K1 supplementation, III—CF patients receiving vitamins K1 and MK-7 supplementation.

**Table 5:** Vitamin K1 concentration and clinical parameters in CF patients.

Clinical Parameters Median (1st–3rd Quartile)		K1 [ng/mL]		
		0.115 (0.069–0.166) (N = 21)	0.763 (0.545–1.571) (N = 21)	*p*
Age [years]		22.1 (20.8–26.0)	23.8 (20.4–31.5)	0.5629
BMI [kg/m^2^]		21.4 (20.4–22.0)	20.6 (19.1–21.6)	0.0919
FEV1 [s]		59.8 (45.4–79.0)	64.0 (55.0–85.1)	0.7444
Vitamin K1 dose [mg/kg/day]		0.023 (0.008–0.054)	0.058 (0.020–0.081)	0.0414
PIVKA-II [ng/mL]		1.97 (0.95–3.37)	1.41 (0.82–3.46)	0.8999
Gender [%]	Female	14 (66.7)	12 (57.1)	0.3757
	Male	7 (33.3)	9 (42.9)	
CFLD [%]	Yes	11 (52.4)	10 (47.6)	0.5000
	No	10 (47.6)	11 (52.4)	
Diabetes [%]	Yes	2 (9.5)	6 (28.6)	0.1190
	No	19 (90.5)	15 (71.4)	
*Ps. aeruginosa* colonization [%]	Yes	13 (61.9)	14 (66.7)	0.5000
	No	8 (38.1)	7 (33.3)	
*CFTR* gene mutations [%]	F508del/F508del	13 (61.9)	7 (33.3)	0.0607
	F508/other or other/other	8 (38.1)	14 (66.7)	
Supplementation of vitamin MK-7 [%]	Yes	7 (33.3)	4 (19.0)	0.2420
	No	14 (66.7)	17 (81.0)	

BMI—body mass index, FEV1—forced expiratory volume in one second, PIVKA-II—undercarboxylated prothrombin, CFLD—cystic fibrosis-associated liver disease.

**Table 6:** Vitamins MK-4 and MK-7 concentrations and clinical parameters in CF patients.

Clinical Parameters Median (1st–3rd Quartile)		MK-4 [ng/mL]			MK-7 [ng/mL]		
		0.459 (0.327–0.585) (N = 21)	1.306 (1.104–1.594) (N = 21)	*p*	0.084 (0.070–0.093) (N = 21)	0.386 (0.259–0.506) (N = 21)	*p*
Age [years]		22.1 (18.9–23.8)	26.1 (18.7–31.5)	0.4504	23.4 (21.9–29.3)	21.3 (18.7–26.8)	0.1218
BMI [kg/m^2^]		21.4 (20.4–22.0)	20.5 (18.8–22.3)	0.1743	21.3 (20.3–22.4)	20.3 (18.5–22.0)	0.0663
FEV1 [s]		71.0 (53.7–80.5)	71.0 (56.0–85.1)	0.6294	64.0 (45.4–79.0)	65.6 (57.0–76.0)	0.6765
Vitamin K1 dose [mg/kg/day]		0.043 (0.009–0.061)	0.054 (0.018–0.081)	0.2224	0.043 (0.007–0.074)	0.016 (0.010–0.030)	0.5044
PIVKA-II [ng/mL]		1.34 (0.61–2.19)	1.41 (0.74–3.14)	0.6689	2.61 (0.90–4.08)	1.78 (0.82–3.46)	0.3786
Gender [%]	Female	15 (71.4)	14 (66.7)	0.5000	13 (61.9)	13 (61.9)	0.6243
	Male	6 (28.6)	7 (33.3)		8 (38.1)	8 (38.1)	
CFLD [%]	Yes	13 (61.9)	7 (33.3)	0.0607	7 (33.3)	9 (42.9)	0.3757
	No	8 (38.1)	14 (66.7)		14 (66.7)	12 (57.1)	
Diabetes [%]	Yes	2 (9.5)	4 (19.0)	0.3314	1 (4.8)	3 (14.3)	0.3030
	No	19 (90.5)	17 (81.0)		20 (95.2)	18 (85.7)	
*Ps. aeruginosa* colonization [%]	Yes	15 (71.4)	12 (57.1)	0.2602	13 (61.9)	12 (57.1)	0.5000
	No	6 (28.6)	9 (42.9)		8 (38.1)	9 (42.9)	
*CFTR* gene mutations [%]	F508del/F508del	12 (57.1)	7 (33.3)	0.1073	10 (47.6)	11 (52.4)	0.5000
	F508/other or other/other	9 (42.9)	14 (66.7)		11 (52.4)	10 (47.6)	
Supplementation of vitamin MK-7 [%]	Yes	6 (28.6)	6 (28.6)	0.6331	4 (19.0)	12 (57.1)	0.0123
	No	15 (71.4)	15 (71.4)		17 (81.0)	9 (42.9)	

BMI—body mass index, FEV1—forced expiratory volume in one second, PIVKA-II—undercarboxylated prothrombin, CFLD—cystic fibrosis-associated liver disease.

**Table 7:** Univariate logistic regression analysis assessing the relationship between serum MK-7 concentrations and all variables.

	r	β	SE	t	*p*
Age [years]	−0.0002	−0.0043	0.1291	−0.0328	0.9740
BMI [kg/m^2^]	−0.0280	−0.1911	0.1267	−1.5078	0.1369
FEV1 [s]	−0.0014	−0.0976	0.1296	−0.7533	0.4546
Vitamin K1 dose [mg/kg/day]	−1.6735	−0.2193	0.1260	−1.7411	0.0868
Vitamin MK-7 dose [µg/kg/day]	0.1237	0.4623	0.1145	4.0387	0.0002
Gender ^a^	0.0231	0.0708	0.1288	0.5500	0.5843
CFLD ^b^	−0.0317	−0.1007	0.1284	−0.7839	0.4362
Diabetes ^c^	0.0356	0.0865	0.1286	0.6728	0.5037
*Pseudomonas aeruginosa* colonization	−0.0406	−0.1247	0.1281	−0.9738	0.3340

SE—standard error, BMI—body mass index, FEV1—forced expiratory volume in one second, CFLD—cystic fibrosis-associated liver disease. ^a^ 0—male, 1—female; ^b^ 0—CFLD no, 1—CFLD yes; ^c^ 0—diabetes no, 1—diabetes yes.

**Table 8:** Multivariate logistic regression analysis assessing the relationship between serum MK-7 concentrations and selected variables.

Clinical Parameters	MK-7 [ng/mL]
*p* model	0.0005
R^2^ for model	0.2266
Adjusted R^2^ for model	0.2004
Vitamin K1 dose [mg/kg/day]	0.326196 {−0.116650 ± 0.117823} ^1^
Vitamin MK-7 dose [µg/kg/day]	0.000491 {0.434790 ± 0.117823}

^1^ *p* {regression slope coefficient ± standard error of regression slope coefficient}.

## Data Availability

The original contributions presented in this study are included in the article, further inquiries can be directed to the corresponding author.

## Abbreviations

CF—cystic fibrosis, BMI—body mass index, FEV1—forced expiratory volume in one second, CFLD—cystic fibrosis-associated liver disease, PIVKA-II—undercarboxylated prothrombin, MK-4 —menaquinone-4, MK-7—menaquinone-7, SE—standard error.
